# Information technology and addiction science: promises and challenges

**DOI:** 10.1186/s13722-021-00216-y

**Published:** 2021-01-26

**Authors:** Nicolas Bertholet, John A. Cunningham

**Affiliations:** 1 Addiction Medicine, Lausanne University Hospital and University of Lausanne, Bugnon 23A, 1011 Lausanne, Switzerland; 2 Center for Addiction and Mental Health, Toronto, ON Canada; 3 Department of Psychiatry, University of Toronto, Toronto, ON Canada; 4 Addictions Department, Institute of Psychiatry, Psychology & Neuroscience, King’s College London, London, UK

**Keywords:** Information technology, Addiction, Commentary, Special issue

## Abstract

**Background:**

Information technology can be used to advance addiction science and clinical practice.

**Main body:**

This special issue, “Information technology (IT) interventions to advance treatment for opioid and other addictions” presents studies that expand our understanding of IT intervention efficacy, patients’ perspectives, and how IT can be used to improve substance use health care and research. This editorial introduces the topics addressed in the special issue and focuses on some of the challenges that the field is currently facing, such as attrition and treatment retention, transferability of intervention paradigms, and the challenge to keep pace with rapidly changing technologies.

**Conclusions:**

Increasing treatment reach is particularly crucial in the addiction field. IT empowers researchers and clinicians to reach large portions of the population who might not otherwise access standard treatment modalities, because of geographical limitations, logistical constraints, stigma, or other reasons. The use of information technology may help reduce the substance use treatment gap and contribute to public health efforts to diminish the impact of substance use and other addictive behaviors on population health.

## Introduction

The first two decades of the twenty-first century have seen the flourishing of digital/information technology healthcare interventions. Information technology (i.e., the use of technology to create, collect, store and exchange information) presents opportunities to improve healthcare through novel interventions, personalization, data management, support for clinicians, and better access to treatment [1–3]. This *Addiction Science & Clinical Practice* special issue advances our understanding of information technology (IT) substance use intervention efficacy, patients’ perspectives, and how IT can be used to improve substance use healthcare and research. This special issue was supported by the NIDA Clinical Trials Network (CTN) Dissemination Initiative.

## Special issue themes

### Data collection and assessment

IT allows the collection of data at times and in ways that might be challenging with conventional tools. Lauckner et al. [4] explored how smartphones and mobile breathalyzers could be used, and showed its feasibility in monitoring alcohol use among patients living with HIV. Ahamad et al. [5] assessed patients willingness to wear an electronic device to detect and alert others in case of opioid intoxication and showed that more than 50 % of participants would be willing to do so.

### Increasing treatment availability

IT allows researchers and clinicians to reach large portions of the population who would not necessarily access standard treatment, because of restrictive access, geographical limitation and stigmatization. McCrabb et al. [6] showed that internet-based interventions for smoking cessation can reach the majority of socially disadvantaged populations. Ekström et al. [7] showed that, while introducing a new perception of distance between the patient and the clinician, internet-based treatment can offer a better sense of confidentiality and that offering diverse content for users to choose from (such as cognitive behavioral therapy modules, therapist support, and discussion forum) is appreciated by patients and may contribute to the attractivity of internet-based treatment [8].

### Intervention efficacy

There is evidence of efficacy for internet-based intervention for alcohol use and smoking but more evidence is still needed for other types of interventions and interventions for other drugs. Notably, while smartphone apps are promising, the review by Colbert et al. [9] shows that current evidence for apps targeting unhealthy alcohol use is still limited compared with available evidence for internet-based interventions. Focusing on cannabis use among adults and adolescents, Sinadinovic et al. [10] showed that a web-based treatment program with therapist guidance was not superior to a waiting list design, although subgroup analyses showed that the intervention may be beneficial for some.

### Development of novel interventions

Research on IT interventions to advance treatment for addictions is very active and novel interventions are being developed and tested. Pedersen et al. [11] present a protocol for a randomized trial for an intervention on alcohol use and risky sex behaviors among college students studying abroad, while Langdon et al. [12] present a protocol on the development of an integrated digital heath intervention to increase patient participation in treatment for opioid use disorder. Palfai et al. [13] studied the perspectives of patients in HIV care in order to develop a tailored intervention targeting chronic pain and heavy drinking and showed that videoconferencing is acceptable to patients.

### Supporting clinicians and researchers in clinical tasks, implementation of treatment, and standardized data capture

Digital tools can help clinicians by eliminating geographical distance and providing standardized and time-efficient tools to conduct clinical tasks. IT represents an opportunity when it comes to implementing substance use screening and substance use disorder assessment and treatment in busy primary care systems. This special series presents notable examples, such as Adam et al. [14] reporting on the feasibility and acceptability of a self-administered electronic screening tool for multiple substance use, and Bart et al. [15] presenting an outline of a clinical decision support for opioid use disorder to facilitate implementation in electronic health records. Electronic health records, as showed by Venkatesh et al. [16], could help opioid use disorder research, but the standardization of data collection and infrastructure is needed to combine different data sources and capitalize on digital data collection to advance clinical knowledge and research.

## Challenges

As interventions are developed and tested, promises may overstate evidence for new technological approaches. We take the opportunity offered by this special issue to focus on some of the challenges the field is facing.

### Attrition and treatment retention

Attrition is a major challenge in assessing the efficacy of digital interventions [17], and is problematic as it introduces bias in scientific evaluations. The limited face-to-face contact offered by digital interventions may encourage people to participate in IT research projects or use digital interventions. But it may also limit the commitment of research participants and lead to high attrition rates. Similarly, the IT field, with its high demands for immediacy and time efficiency, poses great challenges for retaining users in intervention paradigms that may include moments of uneasiness or boredom. Retaining users and research participants will be key to assess which intervention components may be effective and to obtain generalizable results [18].

### Transferability of interventions

While IT offers opportunities to access patients or users in an extended array of contexts, intervention models must be adapted to the digital world. The transferability of interventions developed for the face-to-face clinical context is questionable: some intervention paradigms may not translate well in the digital realm, notably because face-to-face interventions depend on interpersonal interactions and sophisticated language interplay, including non-verbal communication. The various tools/platforms/terminals used to deliver IT interventions all have their specificities with respect to context of use, perception and acceptability. Thus, when changing the format of an intervention, the effects may be different. Transferability of intervention models developed for face-to-face context or other platforms will require the sustained effort of developers to find adapted solutions and will need to be tested for efficacy. In order to engage users, design, usability, and credibility are crucial [18] and could be considered the equivalent of interpersonal skills in the digital world. When developing interventions, these aspects may be as important, if not more, than adding complex content.

### Keeping up with the technological pace

Running a clinical trial—from obtaining funding, developing a digital intervention, to publishing the results—may take years. By the time results are available, significant technological changes could have taken place (e.g. changes in operating systems, design standards, development of new features) and the tested intervention may become irrelevant. Difficulties associated with rapid changes in the technological landscape and with maintaining functioning apps outside of a research project may explain why, among the studied apps identified by Colbert et al. [9], most are already no longer available to the public. As a result, public health and research agencies may be reluctant to invest in rapidly aging technology. Solutions to maintaining an intervention should thus be an integral part of the development of future digital tools.

### Privacy and data protection

Last but not least, technological developments pose significant challenges in terms of privacy and data protection [19, 20]. In order to benefit from the extensive research efforts and data collection that is taking place, rigorous research methods, standardization of research outcomes and digital infrastructure, and privacy will continue to play, a key role in the future development of digital tools in addiction science.

## Conclusions

Information technology offers important opportunities to address addictive behaviors: IT allows researchers and clinicians to reach large portions of the population who would not necessarily access standard treatment, because of restrictive access, geographical limitation and stigmatization. While access to interventions or treatment increases through IT, some parts of the population will have limited to no access to digital resources and thus other options will have to be developed for this population. Future research should also address the fact that retention in IT-based treatment is often lower than in-person environments. Nevertheless, IT has the potential to help reduce the substance use treatment gap and participate in public health efforts to diminish the impact of substance use and other addictive behaviors on population health.

## Data Availability

Not applicable.
